# Identification of genes down-regulated during lung cancer progression: A cDNA array study

**DOI:** 10.1186/1756-9966-27-38

**Published:** 2008-09-15

**Authors:** Mara Campioni, Vincenzo Ambrogi, Eugenio Pompeo, Gennaro Citro, Mauro Castelli, Enrico P Spugnini, Antonio Gatti, Pierluigi Cardelli, Laura Lorenzon, Alfonso Baldi, Tommaso C Mineo

**Affiliations:** 1 Department of Biochemistry and Biophysics "F. Cedrangolo", Section of Pathology, Second University of Naples, Naples, Italy; 2 Department of Thoracic Surgery and Department of Anaesthesiology, Tor Vergata University, Rome, Italy; 3 S.A.F.U. Department, CRS, "Regina Elena" Cancer Institute, Rome, Italy; 4 Experimental Oncology Department, "Regina Elena" Cancer Institute, Rome, Italy; 5 Department of Surgery "A", Second Faculty of Medicine, "La Sapienza" University, Rome, Italy

## Abstract

**Background:**

Lung cancer remains a major health challenge in the world. Survival for patients with stage I disease ranges between 40–70%. This suggests that a significant proportion of patients with stage I NSCLC may actually be under-staged.

**Methods:**

In order to identify genes relevant for lung cancer development, we carried out cDNA array experiments employing 64 consecutive patients (58 men and 6 women) with a median age of 58 years and stage 1 or stage 2 non-small-cell lung cancer (NSCLC).

**Results:**

Basic cDNA array data identified 14 genes as differentially regulated in the two groups. Quantitative RT-PCR analysis confirmed an effective different transcriptional regulation of 8 out of 14 genes analyzed. The products of these genes belong to different functional protein types, such as extra-cellular matrix proteins and proteases (*Decorin *and *MMP11*), genes involved in DNA repair (*XRCC1*), regulator of angiogenesis (*VEGF*), cell cycle regulators (*Cyclin D1*) and tumor-suppressor genes (*Semaphorin 3B*, *WNT-5A *and retinoblastoma-related *Rb2/p130*). Some previously described differences in expression patterns were confirmed by our array data. In addition, we identified and validated for the first time the reduced expression level of some genes during lung cancer progression.

**Conclusion:**

Comparative hybridization by means of cDNA arrays assisted in identifying a series of novel progression-associated changes in gene expression, confirming, at the same time, a number of previously described results.

## Introduction

Lung cancer remains a major health challenge in the world. Despite improvements in staging and the integrated application of surgery, radiotherapy, and chemotherapy, the 5-year survival rate for individuals with lung cancer is only about 15% [1]. Histologically, 80% of the lung cancers are diagnosed as non-small-cell lung cancer (NSCLC), whereas the remaining 20% of cases are diagnosed as small-cell lung cancer (SCLC). On the basis of cell morphology, adenocarcinoma and squamous cell carcinoma are the most common types of NSCLC. The current staging system for NSCLC is based upon the size and location of the primary tumor (T), the involvement of regional lymph nodes (N), and the presence of distant metastases (M) [1]. The standard treatment of patients with stage I NSCLC (T1-2, N0, M0) is resection of the primary tumor alone (no adjuvant therapy) [2]. Survival for patients with stage I disease ranges between 40–70%, and the failure is due to distant recurrences [3]. This suggests that a significant proportion of patients with stage I NSCLC may actually be under-staged. Therefore, if correctly identified, these patients may benefit from adjuvant therapy in addition to resection, with a predictable improvement in the survival rates. Indeed, to identify patients with stage I NSCLC who might benefit from adjuvant therapy, investigators have attempted to identify factors predicting poor prognosis. These studies included analysis of performance status, histologic subtype, size of the primary tumor, the degree of tumor differentiation, mitotic rate, and evidence of lymphatic or vascular invasion [4-8]. However, all of these factors have failed, to date, to precisely identify a group of stage I patients who would benefit from adjuvant therapy. Cigarette smoking remains the main risk factor for lung cancer, accounting for about 90% of the cases in men and 70% of the cases in women [9]. Our research group has investigated in the last years the possible involvement of several molecular mechanisms, such as cell cycle and apoptosis regulators, oncogenes and tumor suppressor genes, cell adhesion molecules, in the pathogenesis and progression of lung cancer [10-19].

In this study, we utilized the cDNA array technique to identify genes differently expressed in patients at early stage of NSCLC.

## Materials and methods

### Patient selection

Table 1 summarizes the characteristics of the patients enrolled in the study. Subjects selected for the analysis were 64 patients, consecutively treated at the Department of Thoracic Surgery of the Tor Vergata University in Rome, who had radical surgery for NSCLC at pathological stage 1 or 2. The study project was submitted and approved by the Human Tissue Use Committee of the University. The patients were staged according to operative and pathological findings based on AJCC/UICC-TNM classification and stage grouping [17]. N-factor was assessed on lymph nodes removed during routine mediastinal lymphadenectomy. A preoperative staging computed tomography (CT) scan was performed in all patients. Histology grading and N-stage were performed on haematoxylin-eosin stained sections. In 22 patients the neoplasm was resected by pneumonectomy and in 42 by lobectomy. Patients who did not survive beyond 60 days after surgery were not included in the study to avoid bias from peri-operative death. Patients who underwent minimal resection were ruled out from the present analysis. Another mandatory prerequisite, was the lack of chemo or radiotherapy before and after surgical resection.

**Table 1:** Patients' Characteristics

*Total number*	64
*Median age*	58
*Male vs Female*	58 *vs *6 (90.5% *vs *9.5%)
*Neoplasm histotype*	
Squamous cell carcinoma	33 (51.5%)
Adenocarcinoma	23 (36%)
Others	8 (12.5%)
*Clinical Stage*	
I	42 (65.5%)
II	22 (34.5%)
*Grading*	
1–2	38 (59.5%)
3	26 (40.5%)
*Surgery*	
Pneumectomy	22 (34.5%)
Lobectomy	42 (65.5%)

### RNA extraction

Total RNA from frozen tumour tissues was extracted utilizing the Atlas™ Pure Total RNA Extraction System (CLONTECH). RNAs were quantified spectrophotometrically and their integrity confirmed by fractionation of 1 μg of RNA on 1% agarose gel with ethidium bromide staining. Then, two populations of RNAs were prepared, including exactly 1 μg of RNA from each patients, separating patients in stage 1 and stage 2.

### Array hybridization

We prepared cRNA for hybridization using the Atlas™ Pure Total RNA labeling System (Clontech). The Clontech Atlas™ Human Cancer Array, composed of 588 human cDNAs, was used for hybridization according to the manufacturer's instructions. Arrays were scanned using a PhosphorImager and analyzed by ImageQuant 5.0 software (Molecular Dynamics, Sunnyvale, California). The experiment was performed in duplicate, utilizing two different RNA preparations. Intensity values were normalized using the expression levels of the housekeeping genes spotted on the arrays. A cut-off of two folds was used to select the genes differentially expressed.

### Quantitative real-time reverse transcription-PCR

The primer sequences are shown in table 2. Primers were designed using Primer Express 2.0 software (Applied Biosystem, Foster City, CA, USA). The specificity of each target amplicon was assessed by dissociation curve analysis and all amplicons were spanning over exon-exon regions to avoid genomic amplification. qPCR was performed on an ABI PRISM 7900HT Sequence Detection System (Applied Biosystems, CA, USA) in 384 well plates assembled by Biorobot 8000 (Qiage**n**, Hilden, Germany) using a final volume of 20 μl and the following cycle conditions: 50°C for 2 min., 95°C for 10 min., and then 40 cycles of 15 s at 95°C and 1 min at 60°C. All QPCR mixtures contained 1 μl of cDNA template (corresponding to 20 ng retro-transcribed totRNA), 1× Sybr Green PCR Master Mix (2×) (Applied Biosystems, CA, USA) and 150 μM of each target-specific primer. For each experiment a no-template reaction was included as negative control. Results have been analyzed using the Applied Biosystems analysis software and expression levels calculated from a linear regression of the standard curve. Results are given as gene target expression vs GADPH expression (gene target relative expression) to correct for differences in the quantity of cDNA used in the PCR reaction. All real-time PCR reactions for each sample were performed in triplicate.

**Table 2:** Expression patterns obtained by array hybridization and confirmation of differential gene expression by Quantitative RT-PCR

*Gene*	*Ref seq ID*	*Array ratio Stage 1/stage 2*	*Confirmed by QPCR*
Cyclin D1	NM_053056	2.04 down	Yes
VEGF	NM_003376	2.02 up	Yes
WNT-5A	NM_003392	2.03 down	Yes
MMP11	NM_005940	3.68 up	Yes
Rb2/p130	NM_005611	3.24 down	Yes
Semaphorin	NM_004263	3.43 down	Yes
Decorin	NM_001920	2.16 up	Yes
XRCC1	NM_006297	3.40 down	Yes

## Results

**Figure 1:**
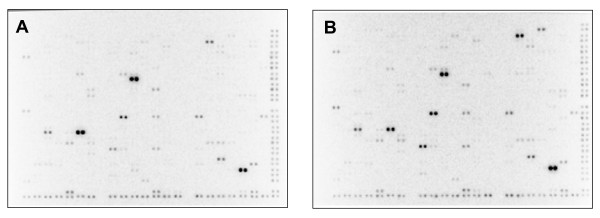
**DNA Arrays: **PhosphorImager output of the two Clontech Atlas™ Human Cancer cDNa Expression Array nylon filters after hybridization with ^32^P-labeled cDNA derived from total RNA from either NSCLC patients at stage 1 and stage 2 (Panels A and B, respectively).

A typical representation of the results obtained hybridizing two identical filter arrays with cDNA-labeled probes generated from either patients in stage 1 and patients in stage 2 is shown in Figure 1, Panels A and B. Computer-assisted analysis of these filters allowed, after normalization, to identify 14 genes, out of 588, as differentially regulated in the two groups (data not shown). To confirm these changes in expression patterns, the genes were further analyzed by Quantitative RT-PCR, using two sets of independently prepared RNAs. Only 8 out of 14 of the genes analyzed were confirmed to be differentially regulated by quantitative RT-PCR. In table 2 are depicted the genes whose differential expression was confirmed with quantitative RT-PCR. In table 3 the primers utilized for RT-PCR are shown. These genes code for different protein families: extra-cellular matrix proteins and proteases (*Decorin *and *MMP11*), genes involved in DNA repair (*XRCC1*), regulator of angiogenesis (*VEGF*), cell cycle regulators (*Cyclin D1*) and tumor-suppressor genes (*Semaphorin 3B*, *WNT-5A *and retinoblastoma-related *Rb2/p130*).

**Table 3:** Primers and conditions utilized for the Quantitative RT-PCR

*Gene*	*Sequence*	*PCR product (bp)*	*Annealing Temp.*
*Cyc D1 for*	TGAGGCGGTAGTAGGACAGG	66	60°C
*Cyc D1 rev*	GACCTTCGTTGCCCTCTGT		
			
*VEGF for*	GCAGTAGCTGCGCTGATAGA	119	60°C
*VEGF rev*	CCTTGCTGCTCTACCTCCAC		
			
*WNT-5A for*	CAAAGCAACTCCTGGGCTTA	107	60°C
*WNT-5A rev*	GCTCGGATTCCTCGGCT		
			
*MMP11 for*	CAGCCAAAGAAGTCAGGACC	147	60°C
*MMP11 rev*	GGTGCCCTCTGAGATCGAC		
			
*Rb2/p130 for*	AGGAGTTTCTCCTGTGCGTATC	106	60°C
*Rb2/p130 rev*	TGAAACAATGCTTTCTCCTCG		
			
*Semaphorin for*	TCACAGTGGCAGCATAGAGG	111	60°C
*Semaphorin rev*	TCACAGTGGCAGCATAGAGG		
			
*Decorin for*	TGCAGGTCTAGCAGAGTTGTGT	91	60°C
*Decorin rev*	AATGCCATCTTCGAGTGGTC		
			
*XRCC1 for*	GACACTTACCGAAAATGGCG	110	60°C
*XRCC1 rev*	GACACTTACCGAAAATGGCG		

## Discussion

In the present study, we analyzed two population of NSCLC patients at stage 1 and 2 to screen for tumor progression-associated genes, by means of a cDNA array filter containing 588 different cDNAs. Consistent with previously reported results [20], not all of the differences found by the array were effectively reproducible using another quantitative mRNA technique, such as quantitative RT-PCR. The discrepancies could be explained by methodological limitations of the array technology, where thousands of diverse cDNAs differing exponentially in expression levels are compared by means of a single hybridization reaction, irrespective of the optimal range of reaction conditions.

The genes we found reliably differentially regulated in the two RNA populations are known to exert different functions, thus confirming that the biological events taking place during the malignant progression involve several molecular pathways.

The *cyclin D1 *gene codes for one of the cyclins involved in cell cycle regulation; specifically for the G1-S transition. The involvement of cell-cycle regulators in the progression of lung cancer is well documented [21]. This observation, therefore, further support the fact that alterations in cell cycle regulation is one of the earlier steps in the progression of NSCLC.

The *VEGF *gene codes for a glycoprotein with potent angiogenic, mitogenic, and vascular permeability-enhancing activities for endothelial cells. Tumour neoangiogenesis has been recently recognized to be of importance in defining subsets of patients with poor outcome in cancer [22,23]. From that several reports [18,24] have stated that the presence of neoangiogenesis represents a significant factor in terms of overall and disease free survival in lung cancer.

The *WNT-5A *gene is a representative ligand that activates a beta-catenin-independent pathway in the Wnt signalling [25]. In contrast to the transforming members of the Wnt family, shown to be up-regulated in many cancers, the role of Wnt 5a is still controversial. While it has been attributed a tumour suppressor function in some malignancies, there is increasing evidence of promigratory and proinvasive effects in others, mediated predominantly through the planar cell polarity pathway and activation of protein kinase C [26,27]. In our experimental setting, transcription of *WNT-5A *was down-regulated in stage 2 NSCLC, thus underlying a putative tumour suppressor function of its gene product in lung cancer progression.

The *MMP11 *gene belongs to a family of matrix metalloproteinases, proteolytic enzymes that degrade extracellular matrix and promote the local or metastatic potential of carcinoma cells, and whose action is restrained by special inhibitors (metalloproteinase inhibitors) [28,29]. The role of MMP11 protease in lung cancer progression has been clearly defined [28,29].

The *Rb2/p130 *gene codes for one of the retinoblastoma-related proteins. Its role as tumour-suppressor is well documented [30,31], as well as its involvement in lung cancer progression [14]. Our data further suggest that alteration on Rb2/p130 gene expression is involved also in the initial steps of NSCLC progression.

Loss of *Semaphorin-A3F *genes occurs frequently in lung cancer and correlates with advanced stage of disease [32]. Moreover, in lung cancer patients, semaphoring gene loss correlates with advanced disease and increased VEGF binding to tumour cells [33,34]. Our data confirms this phenomenon and correlates it also to the initial step of NSCLC progression.

The *Decorin *gene codes for one of the major extracellular matrix protein which has become the focus of various cancer studies [35]. The exact biological role of decorin in cancer has yet to be clarified; however, several experimental data suggest its involvement in cancer progression and metastatization [36].

The *XRCC1 *gene codes for a base excision repair. Several papers have investigated the association between lung cancer and genetic polymorphisms in XRCC1, suggesting a predisposition to lung cancer development for the patients carrying these gene alterations [37,38]. Interestingly, it has been recently described that XRCC1 transcript abundance levels correlate with cisplatin chemoresistance in non-small cell lung cancer cell lines [39]. To the best of our knowledge, no previous report exists about the involvement of the XRCC1 gene in the early steps of NSCLC progression.

## Conclusion

This study confirms the aptitude of the cDNA array technology in defining molecular pathways involved in NSCLC progression. In addition, our results corroborated previously observed expression patterns of a series of genes, and revealed new genes differentially expressed during NCSLC progression. The role of these newly identified genes is being evaluated in further studies analyzing protein expression pattern and function of the proteins *in vitro *and *in vivo *in lung cancer cells.

## Competing interests

The authors declare that they have no competing interests.

## Authors' contributions

All authors read and approved the final manuscript. MC set up the cDNA array protocols and performed the experiment; VA, EP and AG collected the cancer samples; GC and MC gave advise on the work and helped in the interpretation of the data; EPS, PC and LL performed the RNA ectractions; AB supervised all the work and wrote the paper together with TCM.
